# Supplementary material for: Covid-19 and the quality of life of people with dementia and their carers—The TFD-C19 study
Source: PLoS One. 2022 Jan 19;17(1):e0262475. doi: 10.1371/journal.pone.0262475 (PMC8769363; doi:10.1371/journal.pone.0262475)
Supplement: Attachment — Submitted filename: response to reviewers.docx [file pone.0262475.s001.docx]

Centre for Dementia Studies

Brighton and Sussex Medical School

Trafford Centre for Medical Research

University of Sussex

Falmer

East Sussex BN1 9RY

8^th^ December 2021

Dear Dr Ginsberg

**Re: PONE-D-21-19534**

**Covid-19 and the quality of life of people with dementia and their carers – the TFD-C19 Study**

We would like to thank the reviewers for their comments on our manuscript. We have summarised our response to each comment in the table below

We hope that this meets with your approval

Yours sincerely

**Dr Stephanie Daley**

**Senior Lecturer in Older People’s Mental Health & Education**

**Brighton and Sussex Medical School**

| **Amendment requested** | **Changed made** |
| --- | --- |
| Please ensure that your manuscript meets PLOS ONE's style requirements, including those for file naming. The PLOS ONE style templates can be found at  <https://journals.plos.org/plosone/s/file?id=wjVg/PLOSOne_formatting_sample_main_body.pdf> and  <https://journals.plos.org/plosone/s/file?id=ba62/PLOSOne_formatting_sample_title_authors_affiliations.pdf> | Thank you for this, we have amended the format to follow PLOS ONE style requirements |
| Thank you for stating the following in the Acknowledgments Section of your manuscript: “This study was funded by the National Institute for Health Research (NIHR) Applied Research Collaboration Kent, Surrey, Sussex (grant number N/A), as well as Sussex Partnership NHS Foundation Trust and Brighton & Sussex Medical School”.   However, funding information should not appear in the Acknowledgments section or other areas of your manuscript. We will only publish funding information present in the Funding Statement section of the online submission form. Please remove any funding-related text from the manuscript and let us know how you would like to update your Funding Statement. Currently, your Funding Statement reads as follows:  “This study was funded by the National Institute for Health Research (NIHR) Applied Research Collaboration Kent, Surrey, Sussex (grant number N/A), as well as Sussex Partnership NHS Foundation Trust and Brighton & Sussex Medical School The funders had no role in study design, data collection and analysis, decision to publish, or preparation of the manuscript”. Please include your amended statements within your cover letter; we will change the online submission form on your behalf. | We have remoted the funding text from the main document  There is no change to our funding statement and thank you for making the necessary changes. |
| Please include captions for your Supporting Information files at the end of your manuscript, and update any in-text citations to match accordingly. Please see our Supporting Information guidelines for more information: <http://journals.plos.org/plosone/s/supporting-information>. | Captions for supporting information files have been added |
| The [PLOS Data policy](http://www.plosone.org/static/policies.action#sharing) requires authors to make all data underlying the findings described in their manuscript fully available without restriction, with rare exception (please refer to the Data Availability Statement in the manuscript PDF file). The data should be provided as part of the manuscript or its supporting information, or deposited to a public repository. For example, in addition to summary statistics, the data points behind means, medians and variance measures should be available. If there are restrictions on publicly sharing data—e.g. participant privacy or use of data from a third party—those must be specified. | Thank you in terms of data sharing, Consent was not obtained from participants to share data online. Data will be made available upon reasonable request to the corresponding author |
| Specifically, the Authors performed two independent analyses in which they compared T0 vs T1, and T1 vs T2, without a clear explanation relative to this choice (the Authors reported “due to the temporal differences” as reason to perform two t-tests, but again this is not a clear explanation). I would suggest to use a repeated measure ANOVA in which the comparisons can be performed between the three time points (to, t1 and t2) in the same analysis. If the Authors performed a repeated measure ANOVA, they can introduce demographical information as well as any other factors as covariates. This analysis might sound more in line with the aim of the manuscript (changes in QoL during the pandemic) | Thank you for this useful suggestion. In a major revision we have amended the analyses following the reviewers recommendations. This is detailed in methods (ln 140-167) and findings.  *Summary statistics were generated on demographic information for the person with dementia and family carer. Means and standard deviations were reported for continuous data, frequencies and percentages were reported for categorical data. Categorical data were dichotomised. Total scores for the C-DEMQOL (and its subscales) and DEMQOL-Proxy were calculated according to their original development guidelines. This included the rules for handling missing data.*  *To understand how QoL changed over time, a repeated measures ANOVA was run for each dependent variable with the following variables entered as co-variates: duration in study (days), baseline dementia severity (CDR SOB), sex of the family carer (male vs female), sex of the person with dementia (male vs female), family carer relationship (spousal vs non-spousal), dementia diagnosis (Alzheimer vs non-Alzheimer dementia), living status (co-resident vs non co-resident), age of the person with dementia and age of the family carer.*  *Machly’s test was used to determine whether the sphericity assumption could be held. Violations in sphericity were checked, and if Prob>ChiSq was greater than or equal to 0.05, then sphericity was assumed. Modifications of degrees of freedom were made upon detecting a violation in Sphericity. If the epsilon estimate (ε) >0.75, we used Hunh-Feldt correction, if ε<0.75 then the Greenhouse-Geisser correction was used.*  *Alongside reporting the main effect of time, statistically significant interactions were also reported alongside their effect size (partial eta, η_p_^2^). To assist with the interpretation of these interactions, covariates were entered into appropriate bivariate analysis (t-test, one-way ANOVA), with outcome change scores of the DEMQOL-Proxy and C- DEMOQL (T3-T2 and T2-T1).* |
| My second concern regarded the use of the regression analyses to verify the role of the demographical factors on the main differences. The use of the regression analyses should be carefully considered, because the Authors used demographical information (in other words, no controlled factor) as predictors. Since the nature of their data, a multivariate analyses might be more adapted (as I have previously suggested). Moreover, the use of this analyses was in disagreement with what reported in the limitation of study (linea 299-300): indeed, regression analyses are meant to described inferential relationship. Because of this criticism, the results relative to the role of the demographical components on quality of life cannot be correctly understood. | Thank you again. We have amended the analyses (ln 140-167) to follow the reviewers recommendations. This is detailed in methods and findings. |
| Clarify the concept of quality of life (lines 34-35), since it represents a crucial point in their work ; moreover in lines 35-37, the Authors reported that the previous studies did not adopt well-validated measurements? And if it is the case, why it should be matter? | Thank you for this point, we have added in a definition of quality of life.  *QoL can be defined as the subjective assessment of the position of the individual in their life, in the context to their culture and value system, relating to their goals, expectations and concerns, impacted by their physical and psychological health, level of independence, and social relationships (10). (Ln 37-40)*  We have also amended the text to make clearer that there is a need for condition-specific measures of quality in life in dementia, rather than there being a problem with validated measures per se  *Living with, or caring for somebody with dementia impacts upon all of the factors known to affect QoL, it is likely that Covid-19 and associated restrictions may have been more impactful for this group of people. There is therefore a need to measure the impact of Covid-19, using condition-specific measures of QoL, which are tailored to the challenges and realities of daily life with dementia. (Ln 40-44)* |
| Clarify the aim of the study and predictions, at the end of the Introduction; | Thank you for this point, the study aim has been added  *Our study aim was to understand how COVID-19 had affected the QoL, wellbeing, and care of people with dementia and their family carers. (Ln 56-57)* |
| Improve tables in terms of clarity, avoiding acronyms and underlining significant differences. Also, the order was altered between table 5 and 6; | All tables have been improved in terms of clarity and the order is correct |
| Use graphs to show the differences between the scores reported across the three time points; also, a graphical representation of the time points in relation to the contextual information might help Readers in understanding the timeline; | This information has been added into the manuscript and is shown in Fig 1 and Fig 2. |
| Devote more effort in describing the questionnaires used for their research. For example, what dimensions are measured by DEMQOL_Proxy? Is there any information about statistical validity of this questionnaire? The same information should be reported for all the other measurements. Moreover, what is the difference between the DEMQOL_Proxy and the C_DEMQOL?  Moreover, when the CDR was administered (t0 or t1)? | Thank you for this point, the CDR was administered at all time points. The text has been amended to make clear that all measures were used at each time point, and also the provide more information on each of the measures – eg domains and psychometric properties.(Ln 104-132)  The difference between DEMQOL-Proxy and C-DEMQOL has been made clear in the next; namely that DEMQOL-Proxy is a proxy measure of QoL in the person with dementia (as reported by carer) and C-DEMQOL is a self-report of QoL in family carers of people with dementia (Ln 128) |
| In describing the results, measures relative to the effect size should be reported. Moreover, I encourage the authors to report 95 % CI in their analysis, since no a priori sample size was performed (and this is completely understandable). | Thank you for this point, this has been amended |
| Devote more effort in describing the clinical and social implications from their study. Perhaps, people with dementia and their families live a kind of “self-isolation”, which is not so amplified by the pandemic social restriction | Thank you for this suggestions, we have expanded on this point within the manuscript (Ln 319-435)  *It might also be that the social restrictions associated with the pandemic have been less impactful due to pre-pandemic reduction in social networks and associated impact on QoL (29).*  *However, at a more granular level there were subscales of the C-DEMQOL that did change between specific timepoints. From the pre-pandemic period to the first lockdown, our data suggest that meeting personal needs and the carer-patient relationship significantly declined. This perhaps highlights the impact of adjusting to relatively recent restrictions in terms of the halting of support services and shielding. During the pandemic, from the first to the second lockdown there was no recovery in these subscales but they did not continue to decline. But in this second phase there were new declines in the C-DEMQOL feeling supported subscales. These may represent longer term effects of the pandemic on carer QoL.*  *Our findings suggest that services need to proactively identify people with dementia and their carers who are more likely to be struggling due to the pandemic. The need for services to adapt and provide targeted support to more vulnerable groups of carers is of upmost importance. (Ln 419-435)* |
