# Supplementary material for: Covid-19 and the quality of life of people with dementia and their carers—The TFD-C19 study
Source: PLoS One. 2022 Jan 19;17(1):e0262475. doi: 10.1371/journal.pone.0262475 (PMC8769363; doi:10.1371/journal.pone.0262475)
Supplement: Attachment — Submitted filename: response to reviewers.docx [file pone.0262475.s002.docx]

Centre for Dementia Studies

Brighton and Sussex Medical School

Trafford Centre for Medical Research

University of Sussex

Falmer

East Sussex BN1 9RY

22^nd^ December 2021

Dear Dr Ginsberg

**Re: PONE-D-21-19534**

**Covid-19 and the quality of life of people with dementia and their carers – the TFD-C19 Study**

We would like to thank the reviewers for their comments on our manuscript. We have summarised our response to each comment in the table below

We hope that this meets with your approval

Yours sincerely

**Dr Stephanie Daley**

**Senior Lecturer in Older People’s Mental Health & Education**

**Brighton and Sussex Medical School**

| **Amendment requested** | **Changed made** |
| --- | --- |
| I have only one main concern, that regards the CDR and SF-DEM questionnaires. Indeed, in the revised version of the manuscript, I was not able to find the data about CDR and SF-DEM. Indeed, only the results relative to the QoL – related questionnaires were clearly reported in the Results; so that, the Authors should clarify this point. | Thank you for this very helpful suggestion.  We have removed all of the text related to the SF-DEM (ln 119-124) from the manuscript as you rightly point out it, it is not reflected in the aims or results of the study  We have however kept in the text related to the CDR, as the baseline CDR measurement was used to assess whether dementia severity was associated with change in QoL scores over time. This is also reported in the results section in Table 1: Baseline participant characteristics (n=248)  However, we have moved the CDR related text to the end of the measure section. We have also made the purpose of the using the CDR clearer (as below):  *This measure was used to assess whether dementia severity was associated with change in QoL scores over time (ln 140-141)* |
| Also, figures for these two questionnaires may be used to show the scores in the different time points. | We have deleted the original Figure 1 (timescales) and replaced two condensed figures. The first (F1) for C-DEMQOL-Proxy with dates and change in mean scores, and the second (F2) for C-DEMQOL with dates and change in mean scores |
| Abstract seemed to be too long, and not in line with the Journal’s style. | We have reviewed the journal’s guidance on abstracts (namely 300 words, explain how study was done, summarise most important results and their significance)  The abstract was 306 words, and we have now reduced this to 300 words. We believe that the abstract does follow the journal’s guidance. |
| CDR and SF-DEM should not be described along the two QoL – related questionnaires (i.e., the main outcomes of the study) in the Methods, because they assessed two different components. | As discussed above, we have removed the SF-DEM and have altered the text on the CDR |
| Data about the two QoL-related questionnaire would be shown in different figures, since they were related to different scores, with different minimum/maximum, as well as to two different components. Also, the Authors should devote more effort in describing figures in the captions. Such as: “In Figure X, mean (bar) and standard deviation (vertical line) about the mean score (y-axis) registered in thee time point (T0 = before the pandemic; T1 = after XX months after the pandemic; and T2 = XX) at the XXX questionnaire is shown | We have amended these two figures (F1, F2) as requested. |
| Pag. 14, lines 270-272: this sentence seemed to be not entirely clear; please, rephrase it. | Our apologies for this. We have re-worded as follows:  Between T1 and T2 there was a decline of 0.16 points (95%CI -0.45 to 0.76) and between T2 and T3 there was decline of 0.13 points (95%CI -0.46 to 0.72), neither were statistically significant (ln 260-262) |
| In the Discussion, terms like “significantly different” should be avoided. | We have removed this term throughout the discussion. |
| I am not a native speaker, but I am wondering if gender rather than sex may be more appropriate as label in the manuscript, such as "carer sex" may be "carer gender". | Thank you for highlighting this point, we have changed this term throughout the manuscript |
